# PKCα Induced the Generation of Extracellular Vesicles in Activated Platelets to Promote Breast Cancer Metastasis

**DOI:** 10.7150/ijbs.89822

**Published:** 2024-07-15

**Authors:** Jinghua Zhao, Huan Tian, Xiaona Zhao, Lan Lan, Huanhuan Liu, Yi Sun, Fengyan Yu

**Affiliations:** 1 Dept of Breast Surgery, Yat-Sen Breast Tumor Hospital, Sun Yat-Sen Memorial Hospital, Sun Yat-Sen University, Guangzhou, Guangdong, China.; 2 Guangdong Provincial Key Laboratory of Malignant Tumor Epigenetics and Gene Regulation, Sun Yat-Sen Memorial Hospital, Sun Yat-Sen University, Guangzhou, Guangdong, China.; 3 Dept of Breast Surgery, the Third Affiliated Hospital of Guangzhou Medical University, Guangzhou, Guangdong, China.; 4 School of Life sciences, Guangzhou University, Guangzhou, Guangdong, China.; 5 Department of Plastic Surgery, Sun Yat-sen Memorial Hospital, Sun Yat- sen University, Guangzhou, Guangdong, China.; 6 Department of Breast Surgery, First Affiliated Hospital, Zhengzhou University, Zhengzhou, Henan, China.

**Keywords:** Breast Cancer, PEVs, PKCα, DNM2

## Abstract

Platelet extracellular vesicles (PEVs) play an important role in tumor development. However, the mechanisms underlying their biogenesis have not been fully elucidated. Protein kinase Cα (PKCα) is an important regulator of platelet activation, but the effect of PKCα on EV generation is unclear. We used small-particle flow cytometry and found that the number of PEVs was increased in patients with breast cancer compared to those with benign breast disease. This was accompanied by increased levels of activated PKCα in breast cancer platelets. Treating platelets with the PKCα agonist phorbol 12-myristate 13-acetate (PMA) increased the phosphorylation PKCα and induced PEV production, while the PKCα inhibitor GÖ6976 showed the opposite effects. Notably, incubating platelets from patients with benign tumors with the culture supernatant of MDA-MB-231 cells induced PKCα phosphorylation in the platelets. Mass spectrometry and coimmunoprecipitation assays showed that Dynamin 2 (DNM2), a member of the guanosine-triphosphate-binding protein family, might cooperate with activated PKCα to regulate PEV production by breast cancer platelets. Similar results were observed in a mouse model of lung metastasis. In addition, PEVs were engulfed by breast cancer cells and promoted cancer cell migration and invasion via *miR-1297* delivery. These findings suggested that PKCα cooperates with DNM2 to induce PEV generation, and PEV release might triggered by factors in the breast cancer environment.

## Introduction

Platelets are crucial components for blood clotting and hemostasis. When activated by stimuli, platelets aggregate and adhere to the vascular wall. This is accompanied by the release of granules and cytokines, which participate in hemostasis and tissue repair. Recently, increasing evidence has shown that platelets are involved in cancer progression. Platelets adhering to circulating tumor cells (CTCs) protect them from damage caused by blood shear forces and immune system attacks 1, 2. Meanwhile, the cytokines and granules released by platelets facilitate cancer cell survival and metastasis; promote tumor angiogenesis; and allow cancer cells to colonize, survive, and proliferate in metastatic foci 3, 4. Reciprocally, clinical and experimental studies have shown that tumors can “educate” platelets by increasing the number of platelets, inducing platelet aggregation and thrombosis, and altering the phenotype and RNA profile 4-6. These studies demonstrate the interaction between platelets and cancer cells. However, platelets cannot infiltrate the tumor parenchyma, and the mechanism of interaction between platelets and cancer cells in the tumor microenvironment is not fully understood.

Extracellular Vesicles (EVs) can transfer proteins, DNAs, or RNAs derived from their parental cells to recipient cells and they are recognized as carriers of intercellular communication 7, 8. Based on their biogenesis and size, EVs can be described as larger extracellular vesicles (LEVs, microvesicles), small extracellular vesicles (exosomes), and apoptotic bodies. The majority of LEVs detected in the plasma are derived from platelets, and LEVs derived from platelets (PEVs) have been shown to infiltrate the tumor microenvironment and are involved in the progression of many types of cancers 9. However, the mechanism of PEV generation has not yet been fully elucidated. LEVs are usually thought to arise from the plasma membrane blebbing outward to form budding structures, followed by cytoskeleton contractility and pinching off at the LEV necks, resulting in the release of LEVs 10-12. Hence, the process of LEV biogenesis is related to cell activation, cytoskeleton remodeling, and deformation, which resembles the response of platelets to triggers. Some molecules, such as Rho-associated protein kinases (ROCK), calpain, pyruvate kinase M2 (PKM2), proprotein convertase subtilisin/kexin 9 (PCSK9), in the phosphatidylinositol 3-kinase (PI3K)-Akt/glycogen synthase kinase 3 (GSK3) or Src kinase/mitogen-activated protein kinase (MAPK) signaling pathways have been reported to regulate platelet activation and release 13, 14. PKCα is considered to be one of the critical molecules in platelet activation, and it plays important roles in cytoskeleton rearrangements and granule secretion, but whether it participates in PEV generation is unknown 15, 16. In this study, we demonstrated crosstalk between platelets and breast cancer. PKCα in platelets were activated by the culture supernatant of breast cancer cells. Activated PKCα was conjugated with Dynamin 2 (DNM2), enhanced the generation of DNM2, and facilitated the release of PEVs from platelets, while PEVs transferred *miR-1297* from platelets to breast cancer cells to promote tumor progression. These findings provide an experimental basis for the application of antiplatelet agents in the treatment of metastatic cancer.

## Materials and Methods

### Platelet and PEV isolation and incubation with breast cancer cells

The blood sample required for the experiment were obtained with the approval of the Ethics Committee of Sun Yat-sen Memorial Hospital, Sun Yat-sen University (Approval Number: SYSEC-KY-KS-2023-255). Informed consent was obtained from all the patients. Human platelets were isolated from peripheral venous blood by centrifugation at 200 g for 10 minutes, then 800 g for 2 minutes at 20°C. For murine platelets, blood was collected by retroorbital bleeding and centrifuged at 200 g for 15 minutes. Platelet pellets were washed twice with HEPES-buffered Tyrode's solution (130 mM NaCl, 3 mM KCl, 0.3 mM Na_2_HPO_4_, 12 mM NaHCO_3_, 20 mM HEPES, 5 mM monohydrate D-glucose, 0.5 mM MgCl_2_, pH 7.4) and resuspended in HEPES-buffered Tyrode's solution for further investigation.

Washed platelets were stimulated with 1 U/mL thrombin (Sigma-Aldrich) at 37°C for 15 minutes, followed by centrifugation at 2,000 g for 20 minutes. The supernatant was then filtered through 0.8 µm pore filters (Corning Inc., Corning, NY, USA) to remove cell debris. The filtrate was centrifuged again at 20,000 g for 120 minutes at 4°C to harvest PEVs. The PEVs were resuspended in HEPES-buffered Tyrode's solution for further processing 17, 18.

Washed platelets or PEVs were added to the medium of breast cancer cells at a ratio of 100:1 or 10,000:1, respectively, and incubated for 48 hours before replacing the cells with normal medium 19.

### Small particle flow cytometry

According to previously described protocols, equivalent blood samples were centrifuged at 1,500 g for 10 minutes, followed by centrifugation at 3,000 g for 10 minutes to remove the hemocytes. PEVs were harvested from hemocyte-depleted plasma by centrifugation at 16,000 g for 30 minutes. PEVs were collected and labeled with a specific marker for platelets, anti-human CD41 (303705, BioLegend) or anti-mouse CD41 (133905, BioLegend) antibodies, in the dark for 15 minutes before analysis by flow cytometry (FCM) 20. Some experiments required incubation with corresponding antibodies, such as anti-DNM2 antibodies (ab3457, Abcam), during the co-culturing processes. FITC-labelled reference beads (Megamix-Plus FSC, BioCytex, Marseille) of various diameters (100 nm, 300 nm, 500 nm) were used to set up the lower and upper limits of the gates for quantification of the CD41^+^ PEV population. Data were analyzed using FlowJo software (version 10.0).

### Uptake of PEVs by recipient cells

PEVs labeled with a lipophilic orange fluorescent dye, Dil, were washed and incubated with MDA-MB-231 cells at 37℃ for 2 hours. The cells were then washed with phosphate-buffered saline (PBS) and dissociative PEVs were discarded. The DiI-labeled PEVs endocytosed by MDA-MB-231 cells were observed and photographed under a 63× oil immersion objective using a Zeiss LSM 800 confocal microscope (Carl Zeiss, Oberkochen, Germany).

### Wound-healing and transwell assays

Briefly, monolayers of MDA-MB-231 cells were scratched with sterile 200 μL pipette tips to create artificial wounds for wound-healing assays. The cells were then maintained with Dulbecco's modified Eagle medium containing 1% FBS. The wound-healing process was observed and photographed at various time points. After measuring the wound width at 0 and 24 hours, wound-healing rates were calculated as follows: wound healing rate = [(wound width at 0 hours) - (wound width at each time point)]/(wound width at 0 hours) × 100%.

Cell migration and invasion assays were performed using 24-well plates and 8μm transwell inserts chamber (Corning Life Science, MA, USA). For migration assays, the lower chambers were filled with 600 μl culture media containing 10% FBS as an attractant, and 5×10^4^ cells resuspended in 200 μl serum free culture media in the upper chambers were incubated at 37 C, 5% CO_2_. The protocol of invasion assays was similar to that of migration assays, except for matrigel (50 μl/well, Corning Life Science, MA, USA) pre-coated on the upper chambers' membranes before cells addition. Cells invading the membrane or matrigel were fixed and stained with 0.1% crystal violet solution and imaged in a bright field microscope, then quantified by counting the number of cells in five random fields. The average value was then calculated.

### *In vitro* kinase assays

Briefly, *in vitro* kinase assays were performed using purified recombinant proteins PKCa (ab55672, Abcam) and DNM2 (RBP164, Clound-Clone Corp.). These proteins were mixed in 1× Kinase buffer (9802S, Cell Signaling Technology) containing 25mM Tris-HCl pH 7.5, 10mM MgCl2, 0.1mM Na3VO4, 5mM β-glycerophosphate, and 2mM dithiothreitol. The reaction mixture also contained 100mM ATP (A26209, Sigma-Aldrich). After incubation at 30°C for 30 minutes, the reaction was terminated, and phosphorylation was detected by western blot using the Phospho-(Ser) PKC substrate antibody (2261, Cell Signaling Technology) at a dilution of 1:1000. Malantide (HY-P1597, MedChemExpress) was used as a positive control.

### Quantitative real-time PCR and western blotting

Offspring mRNAs were subjected to total RNA extraction using TRIzol reagent (Takara, Tokyo, Japan). The concentration and purity of the extracted RNA were determined using a spectrophotometer (Bio-Rad, Hercules, CA, USA). First-strand complementary DNA (cDNA) synthesis was performed using the PrimeScript^TM^ II 1st Strand cDNA synthesis kit (Takara, Kusatsu, Japan). The synthesized cDNA was diluted in nuclease-free water and stored at -20°C until further analysis. The following primer sequences were used*: GAPDH* forward primer, TTCTTTTGCGTCGCCAGCCGA, and reverse primer, GTGACCAGGCGCCCAATACGA; *PKCα* forward primer, GAAACAAGGCTTCCAGTGCC, and reverse primer, CAGTGTCGGGTCCCTTATCC; *TM4SF1* forward primer, TTCTGGCATCGTAGGAGGTG, and reverse primer, GCCACAGCAGTCATCCTGTT, *DNM2* forward primer, GATCCCGCTGGTCAACAAAC, and reverse primer, CTTCAATCTCCTGCCGGACT.

The miRNA primers were designed and synthesized by Ruibo Biotech Company. The relative gene expression was calculated using the 2^-ΔΔCt^ method, with the reverse transcription products from the control group serving as the calibrator to determine the relative expression level.

Protein abundance was evaluated by western blotting analysis, with normalization to NA-K ATPase or β-actin levels. The primary antibodies used were as follows: anti-NA-K ATPase (ab76020, Abcam), β-actin (8457, Cell Signaling Technology), anti-PKCα (ab32376, Abcam), anti-p-PKCα (ab180848, Abcam), anti-TM4SF1 (ab113504, Abcam), anti-DNM2 (ab3457, Abcam), anti-PTEN (9188, Cell Signaling Technology), anti-E-cadherin (3195, Cell Signaling Technology), anti-SLUG (9585, Cell Signaling Technology), and anti-SNAIL (3879, Cell Signaling Technology). Additionally, antibodies against GAPDH (HRP-60004, Proteintech, Rosemont IL), EXOC3L4 (ab126345, Abcam), SEC10 (ab241472, Abcam), HEAT repeat-containing 5B (ab220780, Abcam), and Sec31A (ab86600, Abcam) were used. Peroxidase-conjugated anti-rabbit (ab6721, Abcam) and anti-mouse (ab6789, Abcam) antibodies were used as secondary antibodies. Chemiluminescence (ECL; Thermo Fisher Scientific) was used to enhance the signals.

### Mass spectrometry

Immunoprecipitated proteins pull-downed by the anti-p-PKCα antibody (ab32376, Abcam) were separated by sodium dodecyl sulfate-polyacrylamide gel electrophoresis (SDS-PAGE), followed by silver staining with a Fast Silver Stain Kit (P0017S; Beyotime, Jiangsu, China). The differentially stained gel bands were excised, destained, reduced, carbamidomethylated, and digested at 37°C overnight with trypsin (sequencing grade, Promega, Madison). Samples were processed using a Q Exative mass spectrometer (Thermo Fisher Scientific) and analyzed against a customized protein database using Mascot v2.3 (Matrix Science, England). Mass spectrometry data were analyzed using Proteome Discoverer (version 1.4, Thermo Fisher Scientific). Quantitative data for each identified protein were processed using Proteome Discoverer as the mean value of the highest peptide ion peak region. Peak lists were searched against the Swiss-Prot database using UniProt.

### Co-immunoprecipitation assay

Equal amounts of platelet membrane proteins were incubated with antibodies against PKCα (ab32376, Abcam), p-PKCα (ab109539, Abcam) or IgG (ab205718, ab172730, Abcam) overnight at 4°C with gentle rotation. A phosphatase inhibitor was added throughout the process. Washed protein A/G magnetic beads were added into each protein sample and incubated for 4 hours at 4°C with gentle rotation. Immune complexes were harvested by boiling the magnetic beads in loading buffer, followed by SDS-PAGE and further analyses.

### Animal experiments

Human breast cancer MDA-MB-231 cells and 6-8-week-old female nude mice were used. All animal experiments were performed according to the National Guidelines for Animal Usage in Research (China), and the protocol was approved by the Ethics Committee of Sun Yat-Sen University. There were four groups of mice, with 10 mice in each group. Luc-labeled MDA-MB-231 single-cell suspensions (2 × 10^6^ cells) were injected into the mice via the tail vein to induce lung metastasis, and an equal volume of PBS was injected into mice in the baseline control group. The experimental mice were then treated with the PKCα agonist Phorbol 12-Myristate 13-Acetate (PMA) (s7791, Selleck Chemicals) at 0.05 mg/kg or the inhibitor GÖ6976 (s7119, Selleck) at 0.4 mg/kg by peritoneal injection every week. Mice in the baseline control group were treated with an equal volume of 0.1% dimethyl sulfoxide (DMSO). Lung metastases were observed weekly via *in vivo* imaging using an IVIS Lumina imaging system (Xenogen IVIS Lumina System, Caliper Life Sciences).

Four weeks after cancer cell injection, the mice were sacrificed. Platelets and PEVs were isolated from the mice and analyzed as described above. After ventricular Dexter perfusion with normal saline three times, the lungs of the mice were excised and the metastatic lesions were photographed using a camera, embedded in paraffin, and serially sectioned. The number of metastatic colonies in the lungs was determined by hematoxylin and eosin (H&E) staining under a bright-field microscope.

## Results

### Platelets promoted the migration and invasion of breast cancer cells and were associated with poor prognosis of breast cancer patients

**Figure 1:**
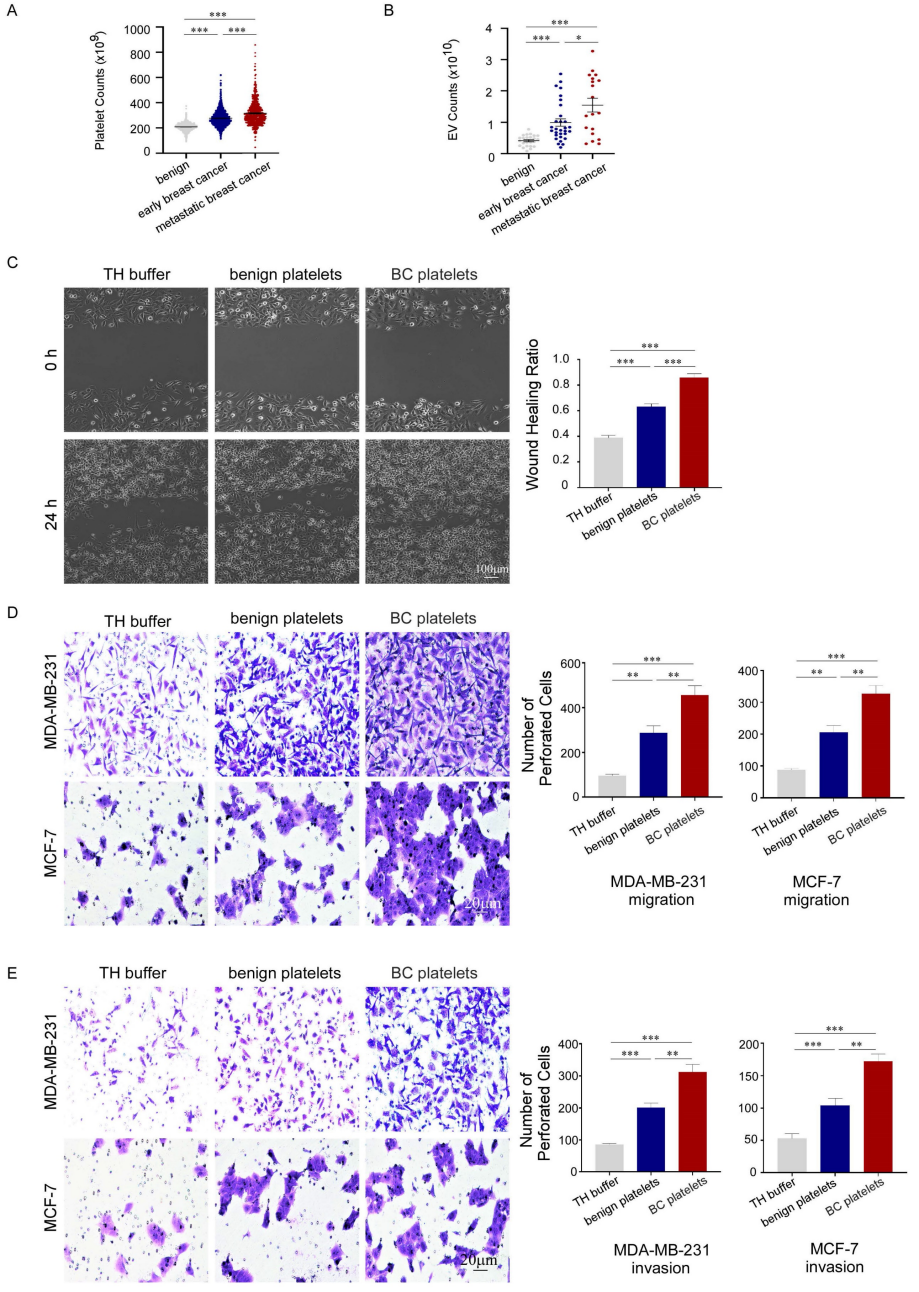
Platelets promoted the migration and invasion of breast cancer cells and were associated with poor prognosis of breast cancer patients. (A) Breast cancer patients had higher platelet counts than benign patients, particularly those with metastatic disease (*p* < 0.001). Data are expressed as the mean ± standard error of the mean (SEM), and were analyzed using a Students' t test. (B) Patients with breast cancer had higher PEV counts than those with benign disease, particularly those with metastatic disease (*p* < 0.001). Data are expressed as the mean ± SEM, and were analyzed using a Students' t test. (C) MDA-MB-231 cells were co-cultured with platelets from patients with breast cancer (BC platelets) or benign patients or with HEPES-buffered Tyrode's solution (TH buffer). A wound-healing assay was performed to evaluate cell migration in the three groups. The wound-healing process was observed and photographed at 0 and 24 hours under 10× magnification, and the unhealed area was measured using Image J software. MDA-MB-231 cell migration was assessed based on the wound healing ratio, which was defined as the proportion of the healed area at 24 hours. Data are shown as the mean ± SEM from at least three independent experiments (*p < 0.05, **p < 0.01, ***p < 0.001). (D, E) The effects of platelets from breast cancer patients or benign patients on the migration and invasion of breast cancer cells were demonstrated by transwell assays (D, MDA-MB-231, upper panel; MCF-7, lower panel) and invasion assays (E, MDA-MB-231, upper panel; MCF-7, lower panel). Three independent experiments were performed and five random microscopic fields were quantified for migratory or invading cells under a bright-field microscope at 20× magnification (*p < 0.05, **p < 0.01, ***p < 0.001).

To investigate the association between platelets and the development of breast cancer, we retrospectively analyzed platelet parameters in routine blood tests from 2,827 breast cancer patients before anti-tumor treatment and 2,825 benign controls (January 2010 to August 2020). The general condition of the cancer patients is presented in supplementary. We found that platelet counts in peripheral blood were significantly increased in patients with breast cancer, and the counts were higher in patients with metastasis than in those with localized cancer (Fig. 1A). Furthermore, we observed a correlation between the number of PEVs and the prognosis of patients with breast cancer (Fig. 1B). To confirm the contribution of platelets to breast cancer progression, we used wound-healing and transwell assays to assess the migration and invasion of breast cancer cells treated with platelets. Platelets accelerated the gap filling of MDA-MB-231 cells in wound-healing assays, and platelets from patients with breast cancer (BC platelets) were more powerful than those from benign platelets (Fig. 1C). Similar results were observed in transwell assays, in which platelets enhanced the migration and invasion of breast cancer cells. In transwell assays, MDA-MB-231 and MCF-7 cells treated with breast cancer platelets exhibited significantly higher infiltration rates than cells treated with benign platelets (Fig. 1D, 1E). These data illustrate that platelets can promote breast cancer cell metastasis, but there are differences between platelets from breast cancer patients and those from benign patients with benign breast disease.

### Platelet activation was boosted in breast cancer patients

As previously reported, after stimulation by different triggers, P-selectin (CD62P) stored in platelet α granules rapidly transfers onto the outer surface of platelet membranes to mediate platelet adhesion to the vascular wall or cells in the bloodstream. Therefore, it is one of the markers of platelet activation. The activated platelets then deform, aggregate, and release granules to participate in coagulation, inflammation, and other activities 21.

**Figure 2:**
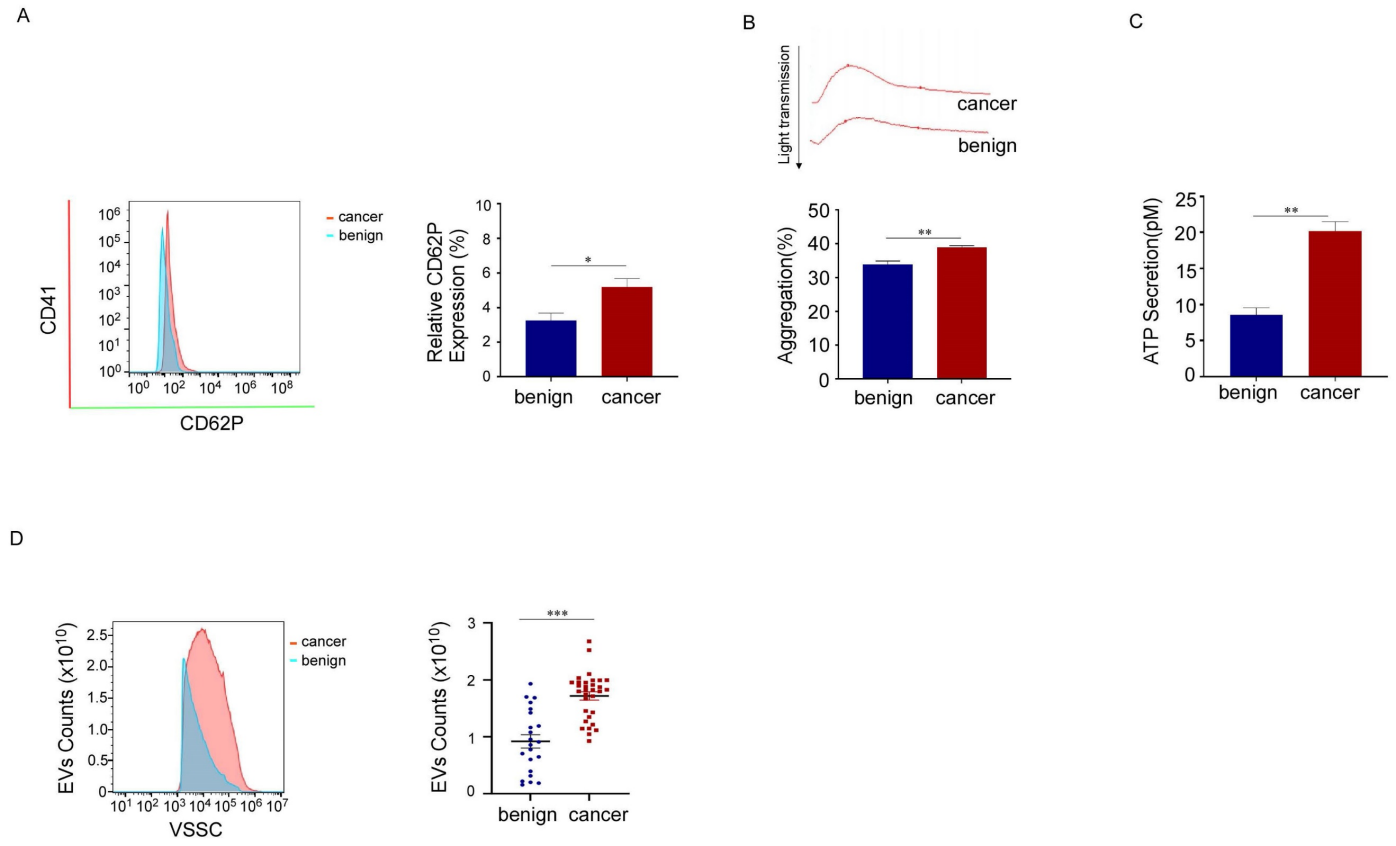
Platelet activation was boosted in breast cancer patients. (A) Representative flow cytometric figure of CD62P expression in platelets from breast cancer patients (n = 35) and benign patients (n = 27). (*p < 0.05, **p < 0.01, ***p < 0.001). (B) Platelets were stimulated with 150 μM adenosine diphosphate (ADP), and then aggregation was detected using turbidimetric aggregometry. Platelet aggregation in response to ADP was enhanced in breast cancer patients (n = 35) compared with benign patients (n = 27). (*p < 0.05, **p < 0.01, ***p < 0.001). (C) Adenosine triphosphate (ATP) release was measured in platelets from patients with breast cancer (n = 35) and benign patients (n = 24). (*p < 0.05, **p < 0.01, ***p < 0.001). (D) The number of PEVs per microliter from patients with breast cancer (n = 32) and benign patients (n = 22) was counted using small-particle flow cytometry based on reference beads, and the results are shown as the mean ± SEM for the number of microparticles. (*p < 0.05, **p < 0.01, ***p < 0.001).

To characterize platelets in patients with breast cancer, we compared CD62P expression levels, platelet aggregation, and the release function of platelets from breast cancer patients and benign patients using flow cytometry, platelet aggregation assays, and ATP secretion detection, respectively. We found that CD62P expression levels, platelet aggregation, and ATP secretion increased in breast cancer platelets (Fig. 2A, 2B, 2C). These data showed that platelet activation is enhanced in patients with breast cancer. As the biogenesis of EVs might be related to cell activation, we also measured the number of PEVs and found that the numbers of PEVs with CD41 (a specific marker for platelets) positivity and diameters ranging from 100 to 500 nm were much higher in breast cancer patients than in benign patients (Fig. 2D). These results suggested that the activation of platelets in patients with breast cancer increases, and the mass production of PEVs might be associated with platelet activation.

### Characterization and function of PEVs

**Figure 3:**
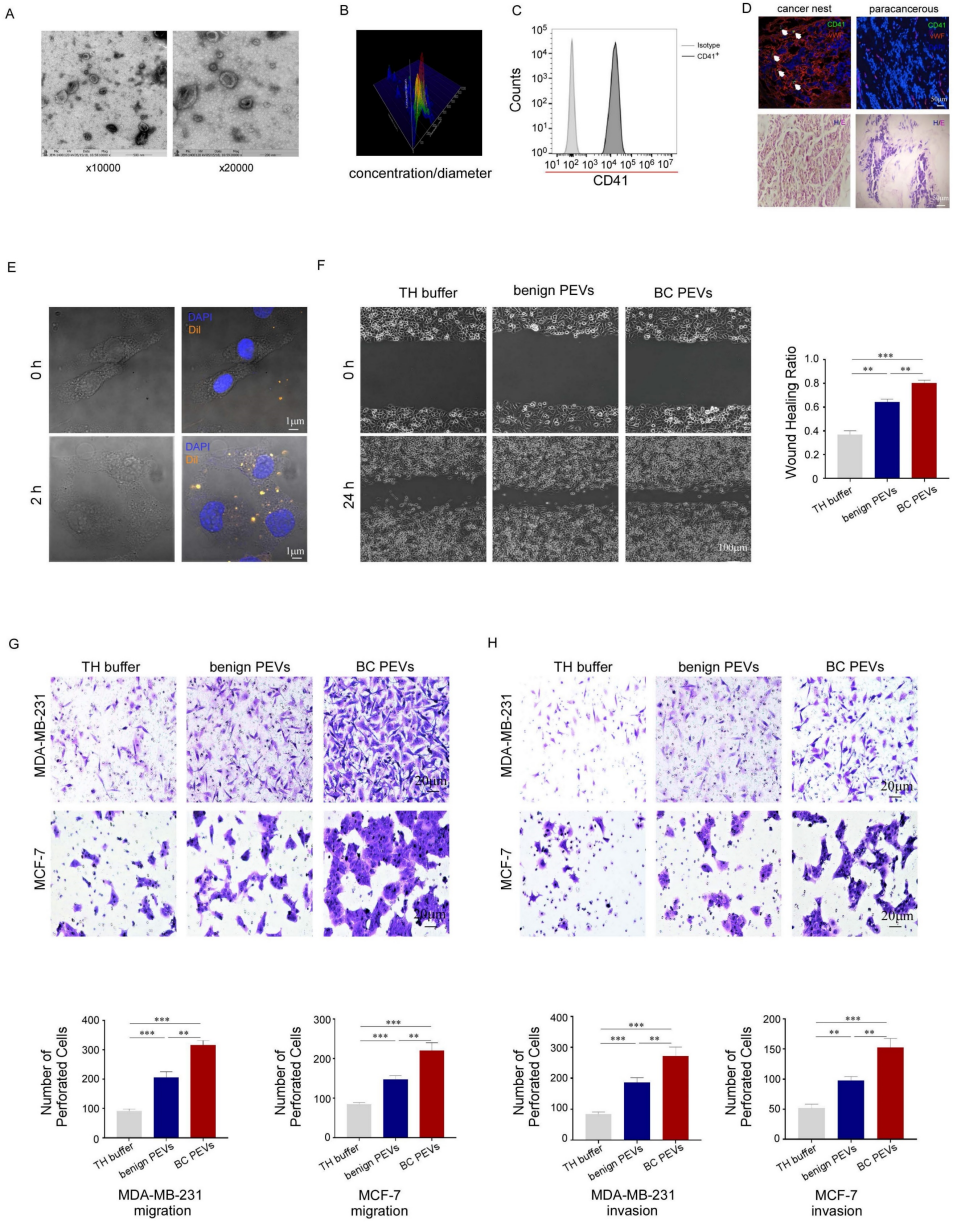
Characterization and function of PEVs. (A) The size and morphology of the PEVs derived from breast cancer platelets were examined by transmission electron microscopy. The PEVs were vesicular bodies with a bilayer membrane structure and diameters ranging from 100 nm to 500 nm. (B) The size and number of PEVs were examined by NanoSight particle size analysis. The diameter of the PEVs ranged from 100 nm to 1000 nm, with most concentrated at 167 nm and the second most at 347 nm. The counts were more than 1 × 10^10^ per microliter. (C) PEVs expressed the platelet-specific protein CD41. (D) Five micron sections from breast cancer tissue and para-cancerous tissue were stained for CD41, von Willebrand factor (vWF, a marker for endothelial cells and platelet function) and with 4′-6-diamidino-2-phenylindole (DAPI). The upper panel shows that PEVs could infiltrate breast cancer tissue, but not para-cancerous tissue. The lower panel showed sections stained with hematoxylin and eosin. (E) Dil-labeled PEVs were phagocytized by MDA-MB-231 cells. The process was photographed at 0 and 2 hours under a 63× oil-immersion objective using a confocal microscope. (F) MDA-MB-231 cells were incubated with PEVs derived from patients with breast cancer (BC PEVs) or benign patients (benign PEVs) or with Tyrode-Hep buffer (TH buffer). A wound-healing assay was performed to evaluate cell migration in the three groups. The wound-healing process was observed and analyzed as described in methods. (*p < 0.05, **p < 0.01, ***p < 0.001). (G, H) The effects of PEVs derived from breast cancer or benign patients on the migration and invasion of cancer cells were demonstrated using transwell assays (G, MDA-MB-231 cells, upper panel; MCF-7 cells, lower panel) and invasion assays (H, MDA-MB-231 cells upper panel; MCF-7 cells, lower panel). Three independent experiments were performed, and five random microscopic fields were quantified for the migratory or invading cells under a bright-field microscope at 20× magnification. (*p < 0.05, **p < 0.01, ***p < 0.001).

Electron microscopy and NanoSight analysis showed that the microparticles in our study had a double-layer membrane and ranged in size from 100 nm to 1,000 nm, with a special pallet shape. Most of the particles fell within the 100-500 nm diameter range, with a concentration greater than 1 × 10^10^ particles per microliter in peripheral blood samples from breast cancer patients (Fig. 3A, 3B). The microparticles were CD41 positive, mirroring the surface markers of their parental platelets (Fig. 3C). Moreover, these microparticles could infiltrate the tumor microenvironment from the dysfunctional tumor vessel wall of breast cancer tissue, but could not extravasate into para-cancerous tissue from intact normal vessels (Fig. 3D). We also found that PEVs labeled with Dil (a lipophilic orange fluorescent dye) could be phagocytosed by MDA-MB-231 cells after 2 hours of coculture *in vitro* (Fig. 3E). Thus, these microparticles are functional PEVs, rather than apoptotic bodies or waste generated by platelets.

Wound-healing and transwell assays were performed to confirm that the PEVs could promote tumor metastasis, similar to their parental platelets. As expected, PEVs accelerated breast cancer cells invasion and migration, filling the gap in a wound-healing assay, and invaded through the membrane, with or without Matrigel, in transwell assays. The effect of PEVs from patients with breast cancer on MDA-MB-231 cells in the wound-healing assay was much greater than that of PEVs from benign patients (Fig. 3F). PEVs from patients with breast cancer also enhanced MDA-MB-231 and MCF-7 cell infiltration in transwell assays (Fig. 3G, 3H), compared to PEVs from benign patients. These data implied that the isolated microparticles in our study were functional PEVs with homologous molecular markers and had similar effects on breast cancer cells as their parental platelets.

### DNM2 was the protein partner of PKCα in PEVs generation

**Figure 4:**
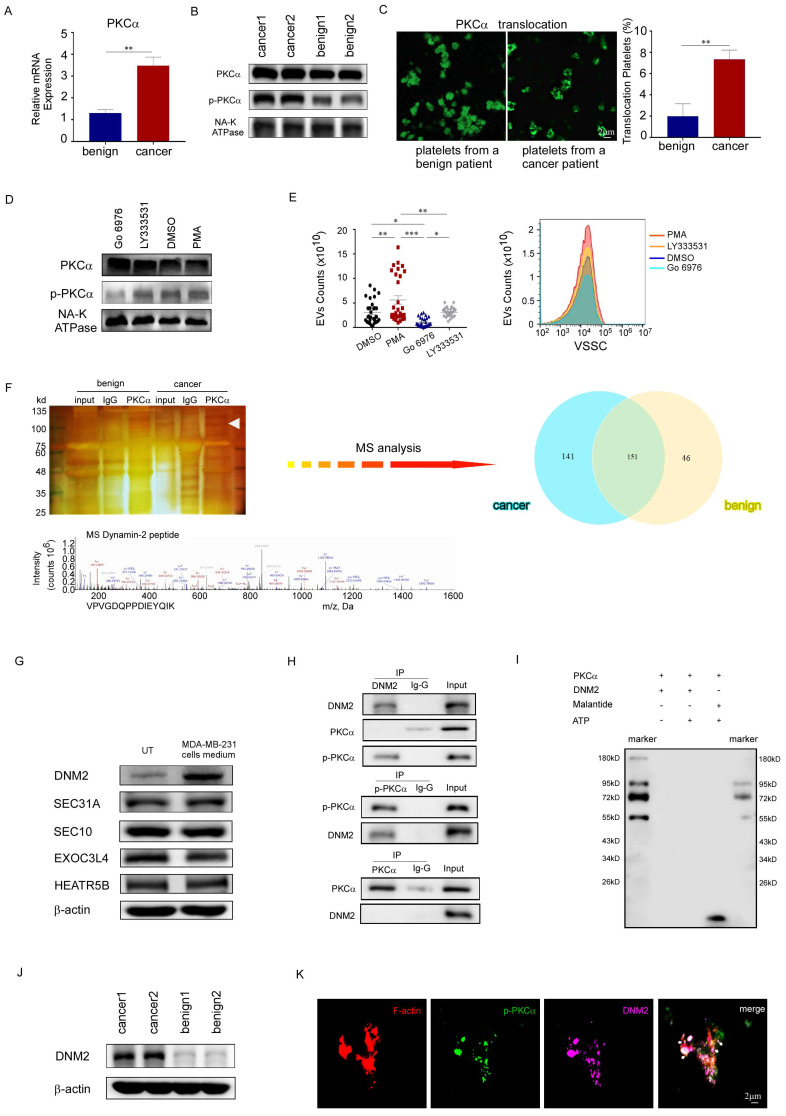
DNM2 was the protein partner of PKCα in PEVs generation. (A) Quantitative reverse transcription polymerase chain reaction (qRT-PCR) was used to quantify the relative expression level of *PKCα* mRNA in platelets from breast cancer patients (n = 3) and from benign patients (n = 3) (*p < 0.05, **p < 0.01, ***p < 0.001). (B) Western blotting analysis of total PKCα and phosphorylated PKCα protein levels in platelets from cancer patients and benign patients. (C) Immunofluorescent staining micrographs show PKCα (green) located in the cytoplasm or membrane of platelets from a benign patient (left), while in platelets from a patient with breast cancer, most PKCα translocated from the cytoplasm to the cell membrane (right). Original magnification of the 63× oil immersion objective in confocal microscopy. (D) Western blotting analysis demonstrated the changes in total PKCα and phosphorylated PKCα protein levels in platelets treated for 30 minutes with 0.05 μM PMA, 1 μM GÖ6976, 10 μM LY333531, or 0.1% DMSO as a vehicle control. (E) Washed platelets from breast cancer patients (n=32) were treated with 0.05 μM PMA, 1 μM GÖ6976, 10 μM LY333531 or 0.1% DMSO for 2 hours. The PEVs in the four groups were then analyzed using small-particle flow cytometry based on reference beads. (*p < 0.05, **p < 0.01, ***p < 0.001). (F) Silver staining of polyacrylamide gels (upper left panel), Venn diagram (upper right panel), and mass spectrometry (MS, lower panel) assays revealed the proteins pulled down from total platelet membrane proteins by an anti-phosphorylated PKCα antibody, along with their overlapping analysis. (G) Western blotting analysis demonstrated that other proteins potentially related to DNM2, including EXOC3L4, SEC10, HEATR5B, and SEC31A, did not show significant changes in response to stimulation in the cancer samples. (H) Co-immunoprecipitation analysis demonstrated that platelet lysates immunoprecipitated with an anti-p-PKCα antibody, but not with an antibody against total PKCα, were DNM2 positive by immunoblotting. Additionally, immunoprecipitation with an anti-DNM2 antibody showed positivity for p-PKCα. (I) *In vitro* kinase assay. ATP was removed from the reaction system as a negative control (lane 2), and compared to the direct substrate of PKCα, malantide (a highly specific substrate for PKCα) (lane 4), used as a positive control. The first and the last lanes were molecular weight marker, indicating the approximate size of proteins in the gel. The results of lane 3 indicated that DNM2 was not a direct substrate of PKCα. (J) Western blotting analysis of DNM2 expression in platelets from cancer patients and benign patients. (K) Platelets from breast cancer patients were stained with phalloidine (red), phosphorylated PKCα (green), and DNM2 (violet). Colocalization of the three proteins was observed and photographed under a 100× oil immersion objective using a confocal microscope. White arrows indicate membrane blebbing and the budding of EVs from platelets, where the three proteins F-actin, p-PKCα, DNM2 colocalized.

Since PKCα had been documented to be an important molecule in the process of platelet activation, we sought to determine whether PKCα was involved in PEV generation in patients with breast cancer. We found that the total PKCα mRNA and protein levels were slightly higher in breast cancer platelets than in platelets from benign patients (Fig. 4A, 4B). However, western blotting analysis showed a significant increase in the phosphorylation level of PKCα in breast cancer platelets compared to its level in normal platelets, and immunofluorescence staining revealed the translocation of PKCα from the cytoplasm to the cell membrane, indicating the activation of PKCα in breast cancer platelets (Fig. 4B, 4C). PMA, a general PKC agonist, induced the phosphorylation of PKCα, and elevated the production of PEVs *in vitro*. Meanwhile, Gö6976, an inhibitor of PKCα and PKCβ, decreased the phosphorylation level of PKCα and reduced the production of PEVs from platelets, compared with the DMSO-treated control group. To determine which PKC subtype was effective, we treated the platelets with the PKCβ-selective inhibitor LY333531 at a maximally effective concentration, and found that LY333531 did not affect PKCα phosphorylation or PEV production (Fig. 4D, 4E). These results suggested the effect of PKCα on production of PEVs from breast cancer platelets.

To identify the protein partner of PKCα in the process of PEV production, we pulled down phosphorylated PKCα protein from platelet membrane proteins using co-immunoprecipitation analysis, with an isotypic IgG antibody as a negative control. The mass spectrometry assay revealed that in the PKCα-containing protein complex, there were 151 similar proteins in platelets from patients with breast cancer and benign patients, while 141 differential proteins were detected only in breast cancer platelets. Bioinformatics analysis showed that 45 of the 141 genes were associated with cell activation signal transmission; 18 were associated with cytoskeleton protein binding or skeletal remodeling; and 8 were associated with vesicle-related events such as production, release, or transport (Fig. 4F). We identified five proteins that may be associated with the generation and release of vesicles in platelets: EXOC3L4, SEC10, HEAT repeat-containing 5B (HEATR5B), Sec31A, and DNM2. However, we found that only DNM2 levels exhibited a significant increase in platelets upon stimulation with MDA-MB-231 cells supernatant (Fig. 4G). Dynamin-2 (DNM2), a member of the GTP-binding protein superfamily, plays an important role in cell activation, cytoskeleton remodeling, and vesicular trafficking 22, 23. Co-immunoprecipitation was performed to investigate the interaction between DNM2 and phosphorylated PKCα. We found that the lysate of platelets immunoprecipitated with an anti-p-PKCα antibody, but not an anti-PKCα antibody, was DNM2 positive by immunoblotting, and those immunoprecipitated with an anti-DNM2 antibody were p-PKCα-positive (Fig. 4H).

PKCα, as a serine/threonine kinase and a member of the protein kinase C family, is extensively involved in various intracellular signaling pathways as it phosphorylates multiple substrates 15. To determine whether DNM2 is a substrate of PKCα, we conducted an *in vitro* kinase assay using recombinant purified proteins for PKCα and DNM2. However, the *in vitro* kinase assay showed that DNM2 was not a substrate of PKCα (Fig. 4I).

We also found the expression level of DNM2 increased in platelets from breast cancer patients, as did p-PKCα levels (Fig. 4J). Furthermore, immunofluorescent staining showed that p-PKCα and DNM2 were co-localized with cytoskeleton protein F-actin on the foci of membrane blebbing during the budding of PEVs from platelets (Fig. 4K). The above-mentioned data suggested that activated PKCα cooperated with DNM2 in facilitating PEV generation from breast cancer platelets.

### Activated PKCα enhanced PEV production and promoted lung metastases in nude mice

To investigate the effect of PKCα on platelet-associated metastasis, nude mice were intravenously injected with MDA-MB-231 cells to establish a lung metastasis model. This was followed by intraperitoneal injection of 0.05 mg/kg PMA, 0.4 mg/kg GÖ6976, or 1% DMSO as a control.

**Figure 5:**
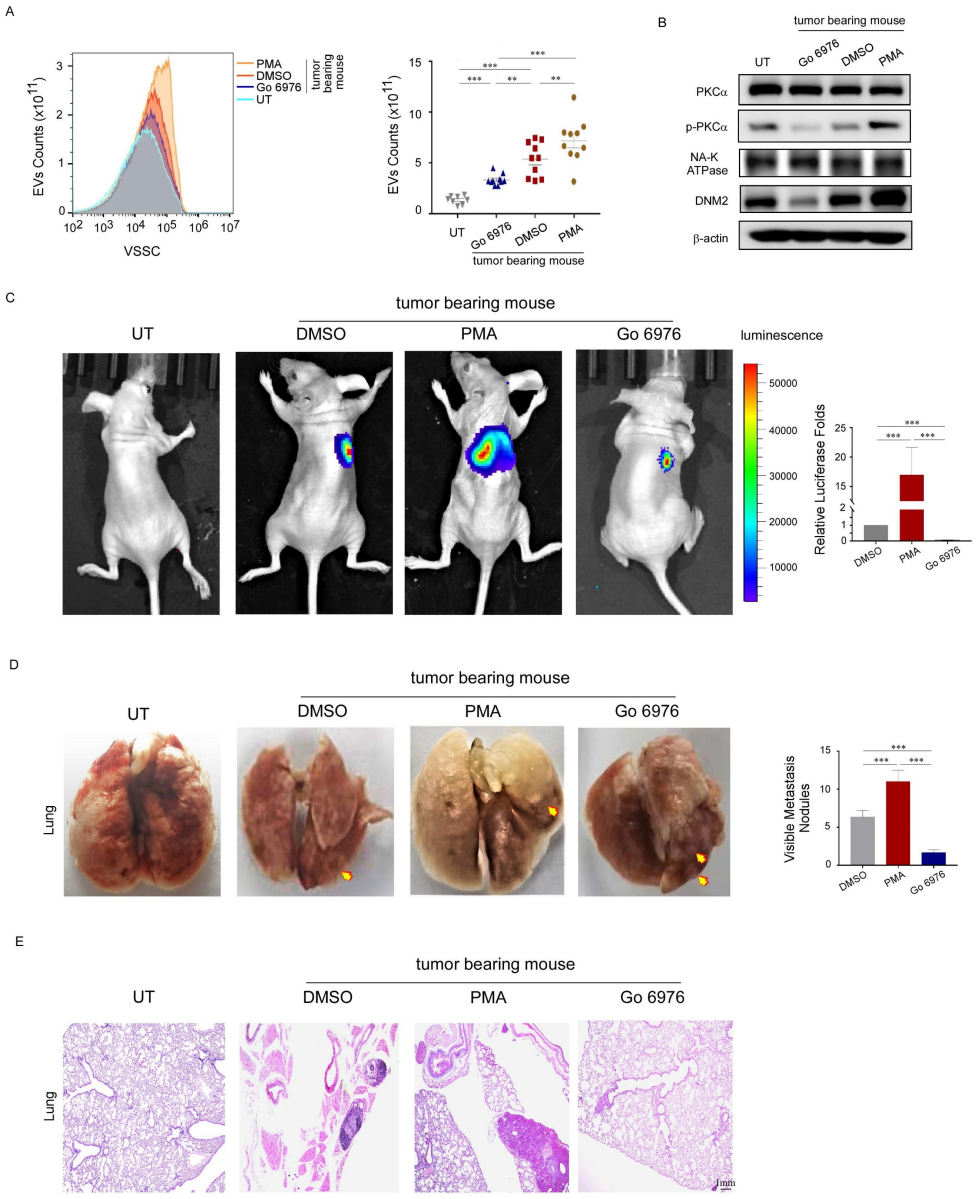
Activated PKCα enhanced PEV production and promoted lung metastases in nude mice. (A) PEVs produced by platelets from tumor-bearing mice treated with PMA, GÖ6976, or DMSO, and from non-tumor-bearing mice were counted using small-particle flow cytometry, and are shown as the mean ± standard deviation for the number of microparticles per microliter. (n = 10) (*p < 0.05, **p < 0.01, ***p < 0.001). (B) Western blotting analysis of PKCα and DNM2 expression in platelets from tumor-bearing mice treated with PMA, GÖ6976, or DMSO, or non-tumor-bearing mice treated with phosphate-buffered saline (PBS). (C) Representative images and semi-quantitative analysis of lung metastases in nude mice using luciferase-based bioluminescence imaging. (*p < 0.05, **p < 0.01, ***p < 0.001). (D) Representative images and quantification of visible metastatic lung nodules in mice from the three groups (yellow arrows). (*p < 0.05, **p < 0.01, ***p < 0.001). (E) Hematoxylin and eosin (H&E) staining of lung metastasis nodes. Original magnification: 10×.

Nude mice injected with PBS instead of MDA-MB-231 cells served as blank controls. As expected, the platelets from nude mice inoculated with MDA-MB-231 cells had higher PEV counts, increased PKCα phosphorylation and DNM2 expression levels. PMA further increased the expression levels of these proteins and the number of PEVs (Fig. 5A, 5B). Moreover, it increased the number and size of pulmonary metastatic foci in mice, as observed using *in vivo* imaging, *ex vivo* visualization, and histological examination (Fig. 5C, 5D, 5E). However, GÖ6976 exhibited the opposite effect. These data demonstrated that activated PKCα enhanced PEV production by mice platelets, and the PEVs were involved in breast cancer metastasis.

### TM4SF1 promoted the increased levels of phosphorylated PKCα and DNM2

**Figure 6:**
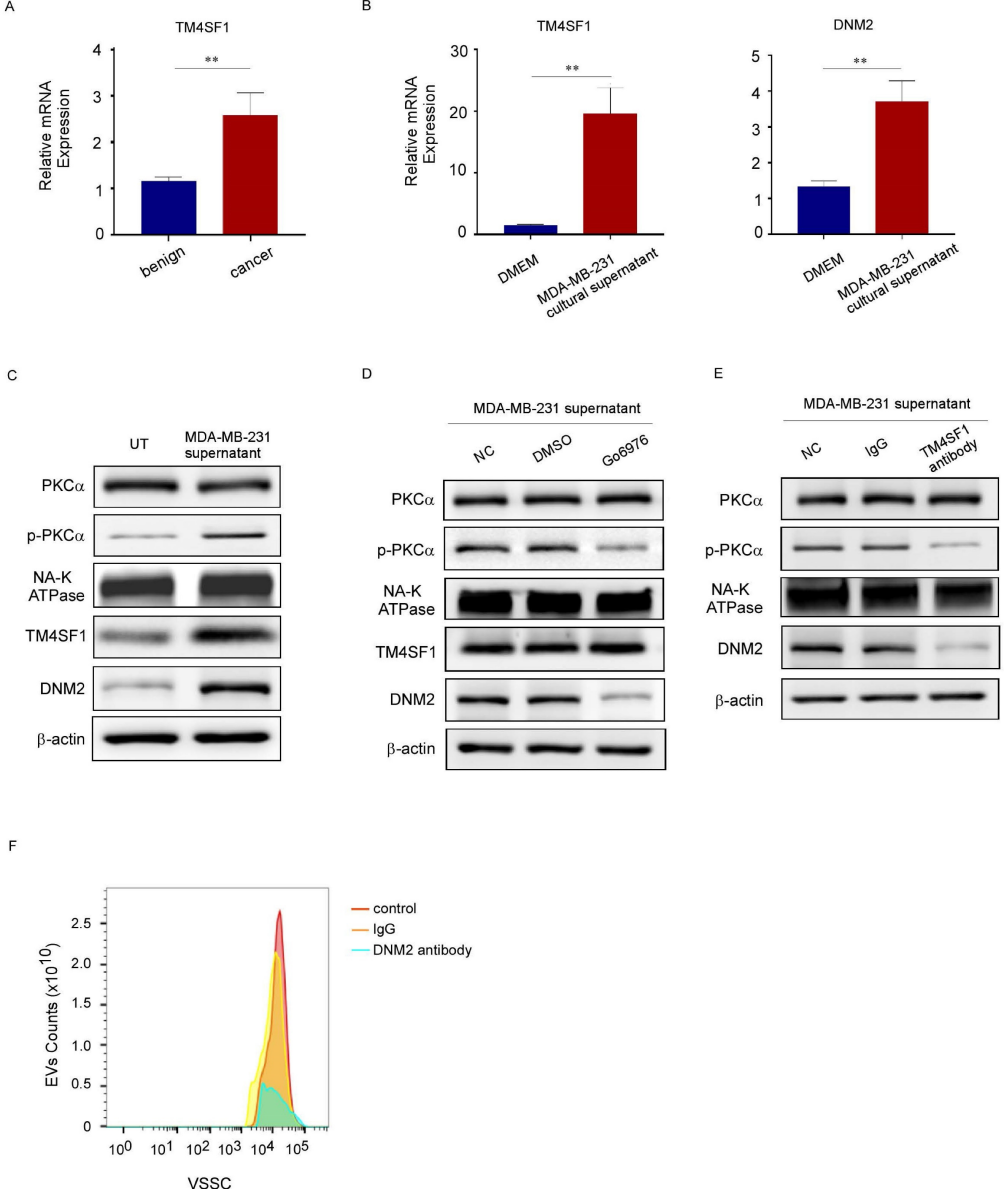
TM4SF1 promoted the increased levels of phosphorylated PKCα and DNM2. (A) qRT-PCR was used to quantify the relative expression level of *TM4SF1* mRNA in platelets from breast cancer patients (n = 3) and from benign patients (n = 3). (*p < 0.05, **p < 0.01, ***p < 0.001). (B) qRT-PCR was used to quantify the relative expression levels of *TM4SF1* and *DNM2* mRNA in platelets treated without or with the culture supernatant of MDA-MB-231 cells. (n = 3) (*p < 0.05, **p < 0.01, ***p < 0.001). (C) Western blotting analysis showed the variation of total PKCα, phosphorylated PKCα, TM4SF1, and DNM2 protein levels in platelets treated with the supernatant of MDA-MB-231 cells for 2 hours, and NA-K ATPase, β-actin were used as loading controls. (D) Western blotting analysis showed the variation in total PKCα, phosphorylated PKCα, TM4SF1, and DNM2 protein levels in platelets treated with the supernatant of MDA-MB-231, and with or without a PKCα inhibitor. (E) Western blotting analysis showed the variation in total PKCα, phosphorylated PKCα, TM4SF1, and DNM2 protein levels in platelets treated with the supernatant of MDA-MB-231 cells, and with or without an anti-TM4SF1 neutralizing antibody. (F) Small-particle flow cytometry showed the variation in PEV counts when treated with an anti-DNM2 neutralizing antibody.

TM4SF1, which is highly expressed in breast cancer, colorectal cancer, and other primary tumors, induces PKC phosphorylation and promotes tumor metastasis 24, 25. Of note, previous studies using mRNA sequencing have reported that the TM4SF1 mRNA level is also increased in platelets from patients with breast cancer 26. Consistent with these reports, our study revealed that *TM4SF1* mRNA levels were elevated in the platelets of patients with breast cancer (Fig. 6A). Besides, treating platelets from benign patients with the culture supernatant of MDA-MB-231 cells increased the mRNA levels of *TM4SF1* and *DNM2* (Fig. 6B), induced PKCα phosphorylation, and increased the protein levels of TM4SF1 and DNM2 in these platelets, but had no effect on total PKCα levels (Fig. 6C). To demonstrate the interaction among TM4SF1, PKCα, and DNM2, we examined the levels of PKCα, p-PKCα, DNM2, and TM4SF1 proteins in platelets from breast cancer patients treated with a PKCα inhibitor or an anti-TM4SF1 neutralizing antibody. The inhibition of PKCα decreased the levels of phosphorylated PKCα and DNM2, but did not affect total PKCα or TM4SF1 levels (Fig. 6D). Meanwhile, the anti-TM4SF1 antibody decreased the level of phosphorylated PKCα, and also reduced DNM2 levels in cancer patients' platelets, but had little effect on total PKCα levels (Fig. 6E). Additionally, in the platelet culture system treated with MDA-MB-231 cells, we added neutralizing antibodies against DNM2 and found that this significantly reduced the release of EVs from breast cancer platelets (Fig. 6F). These results suggested that tumors can alter the mRNA and protein composition of platelets. TM4SF1 induced PKCα phosphorylation, while the latter could interact with DNM2.

### PEVs promoted the metastasis of breast cancer cells by transferring *miR-1297*

**Figure 7:**
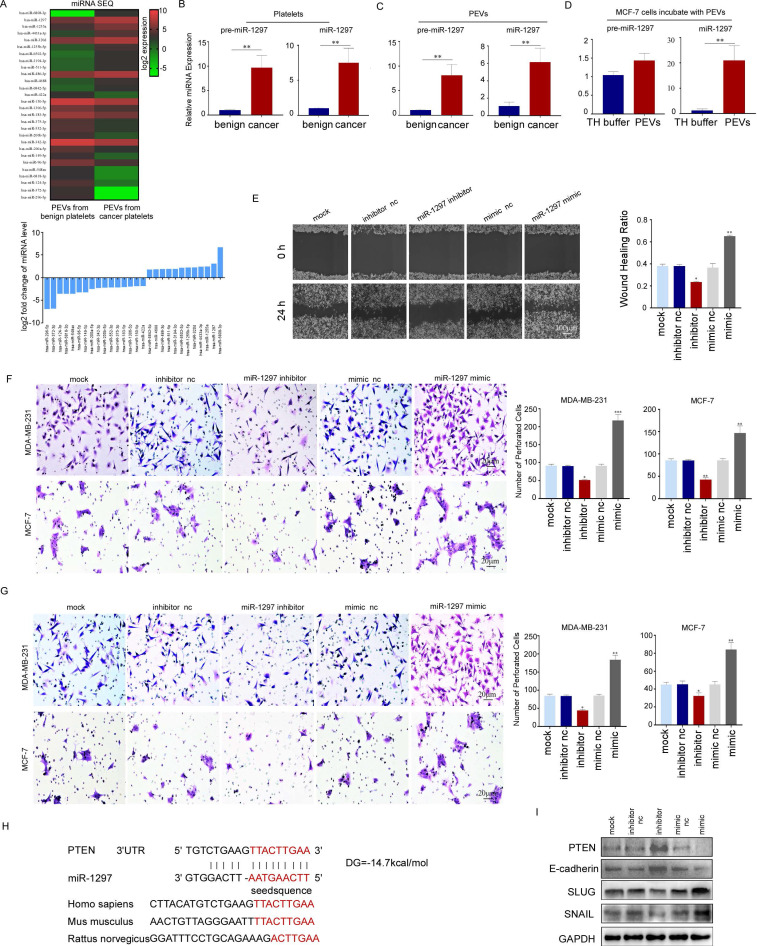
PEVs promoted the metastasis of breast cancer cells by transferring *miR-1297*. (A) Heat map (upper panel) of cluster analysis and histogram (lower panel) showing the relative expression levels of the 14 most abundant and least abundant 18 miRNAs in PEVs from breast cancer platelets using miRNA sequencing. *miR-1297* was one of the most abundant miRNAs. (B) qRT-PCR was used to quantify the relative expression levels of *pre-miR-1297* and *miR-1297* in platelets from patients with breast cancer and benign cancer. (*p < 0.05, **p < 0.01, ***p < 0.001). (C) qRT-PCR was used to quantify the relative expression levels of *pre-miR-1297* and *miR-1297* in PEVs derived from platelets from patients with breast cancer and benign cancer. (*p < 0.05, **p < 0.01, ***p < 0.001). (D) qRT-PCR was used to quantify the relative expression of *pre-miR-1297* and *miR-1297* in MCF-7 cells treated with PEVs derived from patients with breast cancer and with TH buffer. (*p < 0.05, **p < 0.01, ***p < 0.001). (E) MDA-MB-231 cells were transfected with RNase-free water, an inhibitor negative control (nc), a *miR-1297* inhibitor, a mimic negative control (nc), or an *miR-1297* mimic for 48 hours. A wound-healing assay was performed to evaluate cell migration in the five groups. (*p < 0.05, **p < 0.01, ***p < 0.001). (F, G) The effects of *miR-1297* on the migration and invasion of breast cancer cells were demonstrated by transwell assays (F, MDA-MB-231 cells, upper panel; MCF-7 cells, lower panel), and invasion assays (G, MDA-MB-231 cells, upper panel; MCF-7 cells, lower panel). Three independent experiments were performed, and five random microscopic fields were quantified for the migratory or invading cells under a bright-field microscope at 20× magnification. (*p < 0.05, **p < 0.01, ***p < 0.001). (H) Schematic of the predicted binding sites in the *PTEN* 3′-untranslated region (upper) and *miR-1297* (down), and the predicted free energy value of the hybrid is indicated. Seed recognition sites are denoted as red font, and all nucleotides in these regions are highly conserved across species, including humans, mice, and rats. (I) Western blotting analysis showing the variation in PTEN, E-cadherin, SLUG, and SNAIL expression levels in MCF-7 cells transfected with the *miR-1297* inhibitor or *miR-1297* mimic.

miRNA sequencing demonstrated that 12 out of 714 miRNAs were increased by more than three times and 16 were reduced by more than 70% in PEVs derived from breast cancer patients compared with those derived from benign patients (Fig. 7A). Among the 12 upregulated miRNAs, *miR-1297* has been reported to be involved in the proliferation and metastasis of cervical, laryngeal, and other cancers 27-29. We confirmed that *miR-1297* and *pre-miR-1297* levels were increased more than three-fold in PEVs and their parental platelets from breast cancer cells (Fig. 7B, 7C). *miR-1297* levels, but not *pre-miR-1297* levels, were increased in recipient MCF-7 cells incubated with PEVs derived from breast cancer platelets (Fig. 7D). These results implied that the increased levels of *miR-1297* in MCF-7 cells observed in this study were derived from miRNA delivery via PEVs, not from *de novo* miRNA biosynthesis in cells.

To investigate whether exogenous *miR-1297* transferred by PEVs had biological activity in recipient cells, we transfected an *miR-1297* mimic into MDA-MB-231 and MCF-7 cells and found that exogenous *miR-1297* enhanced the migration and invasion of MDA-MB-231 and MCF-7 cells by wound-healing and transwell assays. Conversely, transfection with the *miR-1297* inhibitor suppressed cancer cell motility and invasion (Fig. 7E, 7F, 7G).

Possible target genes of *miR-1297* were predicted using the TargetScan, PicTar, and miRanda databases. We predicted *PTEN* as the target gene because it has been widely demonstrated to be a tumor suppressor in various cancers (Fig. 7H). Furthermore, PTEN expression was downregulated in MCF-7 cells transfected with the *miR-1297* mimic, and this was accompanied by a decrease in E-cadherin expression levels and an increase in the expression levels of SNAIL and SLUG, which play important roles in cell motility. The inhibition of *miR-1297* upregulated the expression of PTEN and E-cadherin, but downregulated the expression of SNAIL and SLUG (Fig. 7I). These results collectively suggest that platelet *miR-1297* can be effectively delivered into breast cancer cells via PEVs and can promote cell migration and invasion by targeting PTEN.

## Discussion

Platelets are involved in tumor metastasis by adhering to CTCs to form clots and release cytokines and granules 1-3. PEVs are granules that are released by activated platelets. High numbers of PEVs are associated with highly aggressive tumors, high tumor loads, and poor prognoses 9, 30. However, the molecular mechanism whereby platelets release PEVs remains unclear. Our findings indicated that PKCα, an essential molecule in the process of platelet activation, was activated in platelets from patients with breast cancer. The collaboration between phosphorylated PKCα and DNM2 played an important role in PEV generation.

PEVs resemble their parental platelets in terms of molecular phenotype and function; however, they can infiltrate the solid tumor microenvironment and can be engulfed by cancer cells to promote tumor development 9, 19. Our findings confirmed the effects of platelets and PEVs on breast cancer metastasis, and the elevated platelet counts and number of PEVs in patients with breast cancer. To explore the mechanism of PEV generation in patients with breast cancer, we characterized platelets from these patients and found that platelet activation was increased. Previous studies have shown that EV biogenesis is modulated by alterations in membrane lipid composition, cytoskeletal remodeling, membrane contractility, and fission. Molecules, including aminophospholipid translocases, scramblases, calpain, small GTPase proteins, and Rho family members, which have been reported to participate in the biogenesis of EVs, also play important roles in platelet activation 10-14. We suspected that molecules involved in the regulation of platelet morphology and function contributed to PEV generation.

PKCα is established as an important molecule in the process of platelet activation and a major regulator of platelet α granule and dense granule secretion. When activated, it is phosphorylated and translocates from the cytoplasm to the cell membrane, where it binds to cytoskeletal proteins to regulate cytoskeletal rearrangements 31, 32. However, the role of PKCα in the generation of PEVs is unclear. Our findings indicated that PKCα was primed in breast cancer platelets. Moreover, treatment of platelets with the PKCα agonist PMA *in vivo* and *in vitro* increased the levels of phosphorylated PKCα and enhanced PEV production, while the PKCα inhibitor GÖ6976 showed the opposite effect. These results demonstrated the role of PKCα in regulating PEV production.

DNM2, a member of the GTP-binding protein family, plays multiple roles in synapse vesicle formation, endocytosis, endosome recycling, and autophagosome precursor release by binding to skeletal proteins and forming rings/helices around the necks of membrane protrusions to promote membrane protrusions and mediate membrane scission 33-35. We demonstrated the interaction between phosphorylated PKCα and DNM2, as well as their effects on PEV generation *in vitro* and *in vivo*. It is speculated that, in the tumor microenvironment, under the influence of different stimuli, phosphorylated PKCα synergizes with DNM2 bound to the cytoskeletal proteins to induce cytoskeletal remodeling, extracellular budding, and PEV formation. Ultimately, DNM2 shears off the PEVs from the neck region of the platelet membranes during budding.

We verified that DNM2 is not a substrate of PKCα, however, due to the short lifespan and cellular instability of platelets *in vivo*, we have not determined the binding sites between phosphorylated PKCα and DNM2, or the specific cleavage site of DNM2 on the platelet membrane during PEV release. Further research is still needed, including bioinformatics analysis and mutant design, to elucidate the interactions among PKCα, DNM2, and other proteins. Additionally, we have not elucidated the transfer process of *TM4SF1* mRNA or *miR-1297*. Fluorescently labeled mRNA or *miR-1297* may be used as probes to demonstrate the mRNA source and delivery process.

We also investigated the mechanism of platelet PKCα activation in the breast cancer environment. Previous studies have shown that tumors can alter platelet function to be more cancer-friendly through various tumor-derived cytokines or by transferring their RNA to platelets 6. In our study, incubating benign platelets with the culture supernatant of MDA-MB-231 cells increased the levels of *TM4SF1* mRNA and activated PKCα in the platelets. TM4SF1 was once reported to be involved in tumor progression and metastasis by promoting the activation of PKCα 26; therefore, we speculated that the TM4SF1 protein derived from breast cancer cells or platelets themselves would induce PKCα activation in platelets. These results suggest complex interactions between tumors and platelets.

We found that PEVs promoted tumor metastasis by transferring their contents, such as *miR-1297*, from breast cancer platelets to breast cancer cells. As high numbers of PEVs are generally associated with a poor prognosis in patients with cancer, and PEVs are not an essential element of blood clotting, they may be the ideal target of antiplatelet agents as an adjuvant treatment for anti-tumor therapy.

In summary, our study provides new mechanistic insights into the crosstalk between breast cancer cells and platelets during tumor progression. Platelets promoted breast cancer metastasis, while platelets themselves could also be “educated” by proteins such as TM4SF1 derived from the tumor to alter their function. PEV release may be a response of platelets to different tumor-derived triggers in the breast cancer environment. They may promote breast cancer metastasis via miRNA delivery. The regulation of PKCα and DNM2 on cytoskeleton protein might be one of the mechanisms of PEV generation.

## Supplementary Material

Supplementary methods.

## Abbreviations

PKCα: Protein Kinase C Alpha
PEVs: Platelet Extracellular Vesicles
EVs: Extracellular Vesicles
LEVs: Larger Extracellular Vesicles
DNM2: Dynamin 2
ROCK: Rho-associated Protein Kinases
PCSK9: Proprotein Convertase Subtilisin/Kexin 9
PI3K: Phosphatidylinositol 3-Kinase
GSK3: Glycogen Synthase Kinase 3
MAPK: Mitogen-Activated Protein Kinase
CTCs: Circulating Tumor Cells
TH buffer: HEPES-buffered Tyrode's solution
FBS: Fetal Bovine Serum
PBS: Phosphate-Buffered Saline
SDS-PAGE: Sodium Dodecyl Sulfate-Polyacrylamide Gel Electrophoresis
FCM: Flow Cytometry
SEM: Standard Error of the Mean
ATP: Adenosine Triphosphate
ADP: Adenosine Diphosphate
qRT-PCR: Quantitative Reverse Transcription Polymerase Chain Reaction
PKM2: Pyruvate Kinase M2
F-actin: Filamentous Actin
TM4SF1: Transmembrane 4 L6 Family Member 1
miRNA: MicroRNA
PTEN: Phosphatase and Tensin Homolog
H&E: Hematoxylin and Eosin
CD41: Cluster of Differentiation 41
P-selectin): CD62P, Cluster of Differentiation 62P
TH buffer: Tyrode's Buffered Solution
E-cadherin: Epithelial Cadherin
SNAIL: SNAIL Zinc-finger Protein
SLUG: SLUG Zinc-finger Protein
