# Supplementary material for: PKCα Induced the Generation of Extracellular Vesicles in Activated Platelets to Promote Breast Cancer Metastasis
Source: Int J Biol Sci. 2024 Jul 15;20(10):3956–71. doi: 10.7150/ijbs.89822 (PMC11302887; doi:10.7150/ijbs.89822)
Supplement: Supplementary file 1 — Supplementary methods. [file ijbsv20p3956s1.pdf]

Patient inclusion and exclusion criteria

Patients in retrospective clinical analysis

This study included patients diagnosed with primary breast cancer histologically confirmed at Sun Yat-Sen Breast Cancer Hospital between January 2010 and August 2020 for a retrospective clinical analysis. Patients were excluded if they had a prior diagnosis of a second primary cancer, a hematological condition, or if they required prolonged use of anti-platelet agents. Age-matched healthy controls for this study were selected from individuals screened at the physical examination center of Sun Yat-Sen Memorial Hospital within the same period (January 2010 to August 2020). The average ages of the healthy controls and breast cancer patients were 44.39 ± 0.22 years and 44.82 ± 0.18 years, respectively, with no significant difference between the two groups ($p > 0.05$).

Characteristics of breast cancer patients

Variables	breast cancer patients
TNM stage	
T1	504
T2	1306
T3	851
T4	114
Indetermination/data missing	52
Grade	
I	149
II	1518
III	1108
Indetermination/data missing	52
ER	
Positive	2146
Negative	599
Indetermination/data missing	82
PR	
Positive	1795

Negative	950
Indetermination/data missing	82
HER2	
Positive	894
Negative	1822
Indetermination/data missing	111

Blood sample and tissue specimens

Blood sample and tissue specimens

Peripheral blood samples and tissue specimens were collected from patients diagnosed with breast cancer or benign breast disease between September 2019 and December 2020, in accordance with the aforementioned inclusion and exclusion criteria, except for the diagnosis of benign breast disease, which was based on histopathological examination. A similar distribution of age was observed in both cohorts.

Characteristics of breast cancer patients

Variables	breast cancer patients
TNM stage	
T1	4
T2	19
T3	10
T4	1
Grade	
I	0
II	29
III	5
ER	
Positive	26
Negative	8
PR	

Positive	25
Negative	9
HER2	
Positive	8
Negative	26

Platelet aggregation

2×10^8 per ml platelets resuspended in Hepes-Tyrode's buffer were treated with 150 μM ADP at 37°C, with continuous stirring at 800 rpm. The platelet aggregation was represented as the percentage change in light transmission during the 3 min with a light scattering platelet aggregometer (BioData Corporation, Horsham, PA).

Platelet immunofluorescence

Washed platelets were inoculated on slides for 6 hours, then fixed, permeabilized, blocked with 5% bovine serum albumin (Thermo Fisher Scientific), and incubated with primary antibody against p-PKCα (ab314198, Abcam), F-actin (Phalloidin, 23119, AAT Bioquest, Pleasanton, CA, USA), DNM2 (sc166526, Santa Cruz Biotechnology) at 4°C overnight. The platelets were then incubated with the appropriate fluorescent-conjugated secondary antibodies (IgG Alexa Fluor Conjugate 649/488, A23610/A23220, Abbkine, Wuhan, China) in the dark at room temperature for 1 hour. During co-culture, a phosphatase inhibitor was added throughout the entire process. Finally, the slides were mounted on coverslips and directly imaged under a 63× or 100× oil immersion objectives using an LSM 800 confocal microscope (Carl Zeiss).

Measurement of ATP secretion

Platelet ATP secretion was monitored using an ATP Assay Kit (Beyotime, China). According to the manufacturer's protocol, platelets were lysed on ice and then subjected to centrifugation at 12,000g for 5 minutes at 4°C. The supernatant was subsequently added to a 100 μl luciferase-luciferin solution. The chemiluminescence

of the samples was quantified using a luminometer (TECAN Spark10M, Switzerland).

Cell lines and culture

The breast cancer cell lines MDA-MB-231 and MCF-7, as well as the embryonic kidney cell line HEK 293T, were purchased from the American Type Culture Collection (ATCC). These cell lines were cultured in either high glucose Dulbecco's Modified Eagle Medium (DMEM) or Roswell Park Memorial Institute (RPMI) 1640 Medium, respectively. Both media were supplemented with 10% EVs-free fetal bovine serum (FBS, System Biosciences, USA). Cultures were maintained at 37°C in a humidified environment with 5% CO2, according to standard protocols. All culture media and related reagents were obtained from Gibco (Invitrogen, USA)

Cell transfection

Cells were seeded in 6 well plates at a density of 1×10^5 cells per well for transient transfection. The following day, cells were transfected with siRNA, miRNA mimic or miRNA inhibitor respectively using Lipofectamine™ 3000 Transfection Reagent (Invitrogen) according to the manufacturer's protocol. Cells were harvested 48–72 hours post-transfection for further experimentation.

Transmission electron microscopy (TEM)

The morphology of PEVs was examined using transmission electron microscopy (TEM). Briefly, PEVs were fixed in 1% glutaraldehyde (Sigma-Aldrich) for 5 minutes. Subsequently, a drop of the PEV suspension was placed onto Formvar-carbon-coated electron microscopy copper grids for 10 minutes. The grids were then stained with 1% uranyl acetate solution (Electron Microscopy Sciences) for 2 minutes. After air drying, the samples were observed and photographed using a JEM-1400 transmission electron microscope (JEOL, Japan).

Nanoparticle tracking analysis (NTA)

The concentration and size of PEVs were tracked by their rate of Brownian motion using the NanoSight NS 300 system (NanoSight Technology, Malvern, UK), and particle-tracking software (NanoSight Version NTA 3.1, Amesbury, UK). After being diluted 10,000 times with filtered PBS, the moving particles were illuminated by the laser, and the rate of PEVs Brownian motion was captured for 60 seconds. The PEVs concentrations and size distribution were calculated using the Nanosight particle tracking software.

Breast tissue stained with haematoxylin and eosin or immunofluorescence (IF)

After deparaffinised, a part of breast tissue sections were stained with haematoxylin for 2 minutes followed by eosin for 1 minute. Other sections were rehydrated, antigen repaired then blocked in 5% BSA, followed by incubation with primary antibody CD41 (MA119381, Invitrogen, USA) and von Willebrand Factor (vWF, PA516634, Invitrogen, USA) at 4°C overnight. Following this, appropriate fluorescent conjugated secondary antibodies (IgG Alexa Fluor Conjugate 555/488, ab150078/ab150113, Abcam) were applied for 1 hour at room temperature. The sections were counterstained with 4′,6-Diamidino-2-phenylindole (DAPI, Beyotime) for 15 minutes, then observed and photographed under a 20× objective using a Zeiss Imager A2 fluorescence microscope (Carl Zeiss, Germany).

RNA-sequencing analysis

RNA was extracted from PEVs using Trizol Reagent (Invitrogen). The purity and quality of the RNA were assessed using a Nanodrop spectrophotometer and agarose gel electrophoresis. miRNA deep sequencing was performed on an Illumina NextSeq 500 sequencer at Kangchen Technology Co., Ltd. (Shanghai, China). The miRNA expression profile data are available in the SRA database under accession number PRJNA841350.

Statistical analysis

All the data as mentioned in the figure legends were presented with error bar as mean ± standard error of mean (SEM) unless otherwise indicated. And data were from a minimum of three independent experiments. Two-tailed unpaired Student's t-test was used for comparing two groups of data and one-way analysis of variance (ANOVA) was for the comparison of multiple groups. Multivariate Cox regression models were used to analyze the correlations between risk factors and survival outcomes, and when $p < 0.05$ the difference was considered statistically significant. Different levels of significance were indicated as *$p < 0.05$, **$p < 0.01$, ***$p < 0.001$. SPSS 22.0 and GraphPad prism 7.0 were used for statistical analyses and scientific graphing.
